# Postoperative Respiratory Depression and Other Postoperative Pulmonary Complications in Veterinary Practice: A Narrative Review

**DOI:** 10.7759/cureus.112870

**Published:** 2026-07-17

**Authors:** Robert B Raffa, Joseph V Pergolizzi, Mohadeseh Abouhosseini-Tabari, Morgan Wagner King

**Affiliations:** 1 School of Pharmacy, Temple University (Emeritus), Philadelphia, USA; 2 Research and Development, Enalare Therapeutics, Mullica Hill, USA; 3 Anesthesiology, NEMA Research, Naples, USA; 4 Shreiber School of Veterinary Medicine, Rowan University, Mullica Hill, USA

**Keywords:** opioid-induced respiratory depression (oird), perioperative monitoring, postoperative pulmonary complications (ppcs), postoperative respiratory depression (pord), veterinary anesthesia

## Abstract

Postoperative respiratory depression (PORD) and other postoperative pulmonary complications (PPCs) represent significant contributors to perioperative morbidity and mortality in companion animal practice. The Confidential Enquiry into Perioperative Small Animal Fatalities (CEPSAF) established that cardiovascular and respiratory causes account for approximately 74% and 72% of perioperative deaths in dogs and cats, respectively, with 47% of canine and 61% of feline anesthetic-related deaths occurring in the postoperative period. PORD arises from a convergence of opioid-induced respiratory depression (OIRD), residual inhalant anesthetic effects, other drug-induced effects (e.g., neuromuscular blockade), and patient-specific vulnerabilities. PPCs encompass a broad spectrum of conditions, including atelectasis, aspiration pneumonia, pneumonia, hypoventilation, acute respiratory distress syndrome (ARDS), and respiratory arrest, with laparotomy studies reporting a PPC incidence of 22% in dogs. This narrative review synthesizes current evidence on the pathophysiology, risk factors, diagnostic approaches, prevention strategies, and treatment of PORD and PPCs in veterinary practice, incorporating insights from both veterinary-specific and translational human anesthesia literature, while explicitly identifying areas where veterinary-specific evidence remains limited. The importance of continuous monitoring during the postanesthetic period, multimodal analgesia to reduce opioid exposure, protective lung ventilation strategies, and early intervention is highlighted as a key strategy to improve outcomes, together with the unmet need for reversal-independent ("agnostic") respiratory stimulants capable of countering PORD irrespective of the causative agent.

## Introduction and background

The perioperative period represents one of the most vulnerable phases in a veterinary patient's clinical course [1]. Advances in anesthetic pharmacology, monitoring technology, and perioperative care have substantially reduced anesthetic mortality in small animals over recent years, but respiratory complications remain among the leading causes of perioperative prolonged morbidity and death [2]. The landmark Confidential Enquiry into Perioperative Small Animal Fatalities (CEPSAF), a prospective cohort study conducted across 117 veterinary practices in the United Kingdom between 2002 and 2004, revealed that anesthetic-related mortality rates in healthy dogs and cats are approximately 0.05% and 0.11%, respectively, rising to 1.33% and 1.40% in sick patients classified as American Society of Anesthesiologists (ASA) grades III-V [3-5].

Two distinct but interrelated phenomena drive respiratory morbidity in the postoperative period: postoperative respiratory depression (PORD) and postoperative pulmonary complications (PPCs). PORD refers (mostly qualitatively to date) to inadequate or impaired respiratory drive and mechanics following surgery and anesthesia, most commonly as a consequence of opioid administration, residual inhalant anesthetic, or neuromuscular blockade [6]. PPCs include a spectrum of pulmonary pathologies that develop following surgery, including atelectasis, aspiration pneumonia, pneumonia, hypoventilation, ARDS, and respiratory failure [7]. PORD and other PPCs contribute to increased oxygen requirements, prolonged ICU stays, and increased morbidity and mortality [8]. Despite the clinical importance of PORD and PPCs, veterinary-specific evidence remains fragmented: most incidence and risk-factor data derive from single-institution retrospective series, and comparatively little prospective or multicenter work has examined monitoring strategies, prevention strategies, or treatment efficacy.

This review aims to provide a concise synthesis of current knowledge on PORD and PPCs within the veterinary context, drawing upon peer-reviewed veterinary literature and translational data from human anesthesiology where species-specific data are limited. Extrapolation from the human literature is used deliberately and cautiously throughout this review. It is noted explicitly wherever a recommendation rests primarily on human data, because differences between species in opioid receptor pharmacology, airway anatomy, body size, and recovery behavior can limit direct applicability, and because thresholds validated in human patients (e.g., neuromuscular monitoring cut-offs) have not been formally verified in animals. Special emphasis is placed on the post-anesthetic recovery period, a phase that the CEPSAF study identified as particularly hazardous, with most deaths occurring within the first three hours following cessation of anesthesia.

Specifically, this narrative review aims to: (1) summarize the pathophysiological mechanisms underlying PORD, including opioid-, inhalant-, and neuromuscular blockade-related respiratory depression; (2) classify the spectrum of PPCs encountered in small animal patients; (3) synthesize veterinary and translational risk factors for PORD and PPCs; (4) review current diagnostic and monitoring approaches during the perioperative and recovery periods; (5) summarize prevention and treatment strategies, including the rationale for reversal-independent ("agnostic") respiratory stimulants as an alternative or adjunct to naloxone in cases where the causative agent is unclear or reversal is undesirable; and (6) identify priority areas for future veterinary-specific research. This scope distinguishes the review from prior veterinary anesthesia overviews by explicitly separating veterinary-validated evidence from translational extrapolation and by identifying the gaps that limit each.

## Review

Materials and methods

This is a narrative, non-systematic review; it does not follow PRISMA reporting standards, was not registered prospectively, and does not include a formal risk-of-bias assessment or PRISMA flow diagram, as none of these are applicable to a narrative synthesis. Reporting was informed by the Scale for the Assessment of Narrative Review Articles (SANRA) recommendations for transparency in narrative reviews.

A literature search was conducted in PubMed/MEDLINE (Bethesda, Maryland) and Google Scholar (Mountain View, California), supplemented by hand-searching the reference lists of retrieved articles and relevant clinical guideline documents (e.g., the American Animal Hospital Association (AAHA) 2020 Anesthesia and Monitoring Guidelines), from database inception through June 2026. Search terms combined controlled vocabulary and free text for the following concepts, joined with Boolean operators: ("postoperative respiratory depression" OR "opioid-induced respiratory depression" OR PORD OR OIRD) AND (dog* OR cat* OR canine OR feline OR veterinary OR "small animal") AND (anesthesia OR anaesthesia OR postoperative OR perioperative OR recovery); a parallel search combined (atelectasis OR "aspiration pneumonia" OR pneumonia OR hypoventilation OR "acute respiratory distress syndrome" OR ARDS OR "pulmonary complication*") with the same species and perioperative terms. Eligible sources were peer-reviewed, English-language publications (original research, clinical reviews, and consensus guidelines) addressing the pathophysiology, risk factors, diagnosis, prevention, or treatment of PORD or PPCs. Veterinary-specific studies in dogs and cats were prioritized; translational literature from human anesthesiology was included only where veterinary-specific data were sparse or absent, and such extrapolation is flagged explicitly at each point of use in the text. Conference abstracts without accompanying full-text publication, non-peer-reviewed sources, and articles not available in English were excluded. Candidate articles were screened for relevance to the review's scope by the author team, with more recent veterinary publications favored over older sources when both addressed the same question; older mechanistic or foundational studies (e.g., early work on inhalant effects on pulmonary host defense) were retained where no more recent veterinary data existed to supersede them. Because the included literature is heterogeneous in design, species, and outcome definitions, no meta-analysis, meta-regression, or other quantitative pooling of effect estimates was performed or was appropriate; findings are instead synthesized narratively, with original numerical results (e.g., incidence rates, odds ratios, and mortality rates) reported directly from the source studies rather than pooled. Claude (Sonnet 4.6, Anthropic, San Francisco, California) was used as a literature-identification and drafting-assistance tool to help identify candidate publications and organize the initial synthesis; all citations and factual claims generated with its assistance were independently verified by the authors against the original source publications, and the authors take full responsibility for the accuracy and interpretation of the content presented.

Results

Pathophysiology of Postoperative Respiratory Depression

Opioids remain the cornerstone of perioperative analgesia in veterinary medicine and are routinely employed in premedication, intraoperative maintenance, and postoperative pain management [9]. Their analgesic efficacy, however, is invariably accompanied by dose-dependent opioid-induced respiratory depression (OIRD) mediated primarily through the mu-opioid receptor (MOR), including activation in the pre-Bötzinger complex of the brainstem, which serves as a central respiratory rhythm generator [10]. Activation of MORs in this nucleus suppresses respiratory rate, decreases tidal volume, and blunts the ventilatory response to hypercapnia and hypoxia [11].

The affinity of individual opioids for MOR significantly influences the reversibility of respiratory depression [12]. Opioids with intermediate binding affinity, such as methadone, fentanyl, and hydromorphone (Ki values of about 1-5 nM), are widely used in veterinary anesthesia and carry meaningful risks of OIRD. High-affinity opioids such as buprenorphine and sufentanil (Ki values < 1 nM) are more resistant to reversal with the MOR antagonist naloxone at equivalent doses because they are displaced less readily from MOR [11]. This distinction is clinically relevant during recovery, when opioid plasma concentrations can persist due to metabolism to active metabolites or redistribution from peripheral compartments back into the central circulation [13].

In dogs, MOR agonists such as morphine, hydromorphone, fentanyl, and methadone provide good analgesia but carry the greatest risk of respiratory depression, particularly in patients with pre-existing respiratory disease, obesity, or brachycephalic anatomy. Cats, owing to species-specific differences in MOR distribution and hepatic metabolism, may exhibit both dysphoric and respiratory depressant responses to opioids [14]. Partial agonists such as buprenorphine and agonist-antagonist drugs such as butorphanol generally produce less profound respiratory depression owing to their ceiling effect on MOR activation, though they might provide reduced analgesia [15]. The concurrent administration of other CNS depressants, including benzodiazepines, alpha-2 adrenoceptor agonists (alpha-2 agonists), and inhalant anesthetics, substantially amplifies the risk of OIRD through additive or synergistic respiratory depression [16].

Volatile inhalant anesthetics, such as isoflurane and sevoflurane, produce dose-dependent respiratory depression through suppression of brainstem respiratory centers and depression of hypercapnic and hypoxic ventilatory drive [17]. Both agents reduce tidal volume and, at deeper planes of anesthesia, respiratory rate. Following cessation of inhalant delivery, recovery depends on elimination through the lungs, a process that is rapid for sevoflurane (with a blood-gas partition coefficient of about 0.6) but somewhat slower for isoflurane (with a large blood-gas partition coefficient of about 1.4), particularly in patients with compromised pulmonary function or prolonged anesthetic duration. During the transition from inhalant anesthesia to room air, patients experience a brief period of reduced hypercapnic drive, which combined with residual opioid effects can precipitate hypoventilation and hypoxemia in the recovery period [18].

Administration of 100% oxygen during recovery, while counteracting hypoxemia, can paradoxically eliminate hypoxic ventilatory drive, delaying respiratory recovery. The AAHA 2020 Anesthesia and Monitoring Guidelines emphasize that positive pressure ventilation during anesthesia, if excessive, lowers end-tidal CO_2_ (ETCO_2_) below the threshold that stimulates spontaneous ventilation, potentially delaying respiratory recovery [19]. Clinicians must balance adequate oxygenation against the risk of suppressing the residual respiratory drive that initiates spontaneous breathing.

Although neuromuscular blocking agents (NMBAs) are used less routinely in general veterinary practice than in human practice, their use in ophthalmic procedures, thoracic surgery, and facilitation of controlled mechanical ventilation introduces the risk of residual neuromuscular blockade (RNMB) at extubation [20]. RNMB occurs when reversal is incomplete, leaving the diaphragm and accessory respiratory muscles with reduced contractile capacity. Quantitative neuromuscular monitoring using "train-of-four" (TOF) ratio assessment is the gold standard for confirming adequate reversal, though its routine use in veterinary practice remains limited [21]. The TOF ratio exceeding 0.9 before extubation is considered safe in human medicine; comparable thresholds have not been formally validated in companion animals, though the physiological principles are the same. Indeed, the absence of TOF fade in cats and dogs does not reliably indicate the restoration of neuromuscular function [22,23].

Postoperative pain, particularly following thoracotomy, laparotomy, or rib fractures, leads to voluntary splinting, whereby patients restrict thoracic expansion to avoid pain during inspiration. The resultant shallow, rapid breathing pattern (tachypnea with reduced tidal volume) promotes progressive microatelectasis and impairs clearance of airway secretions [24]. This creates a paradox in which adequate analgesia is required to facilitate normal ventilation, yet excessive opioid administration risks OIRD. Multimodal analgesia (where species-appropriate) that incorporates regional anesthesia, non-steroidal anti-inflammatory drugs (NSAIDs), and adjuncts such as ketamine or gabapentin can be helpful to balancing the competing demands [25].

Classification and Spectrum of Postoperative Pulmonary Complications

PPCs represent a heterogeneous group of conditions, and their classification in veterinary medicine largely mirrors that employed in human surgery. The major PPCs observed in small animal patients include pulmonary atelectasis, aspiration pneumonia, infectious pneumonia, hypoventilation, hypoxemia, ARDS, and respiratory failure.

Atelectasis is the most prevalent PPC in both human and veterinary surgical patients [26]. It occurs in three forms, based on three principal mechanisms: "absorption atelectasis," "compressive atelectasis," and "passive atelectasis." "Absorption atelectasis" occurs when high concentrations of inspired oxygen (>60%) lead to replacement of nitrogen, a primary stabilizer of alveoli, with oxygen, which is rapidly absorbed into the bloodstream, resulting in alveolar collapse. "Compressive atelectasis" results from mechanical compression of lung parenchyma by mediastinal structures, abdominal contents in dorsal recumbency, pleural effusions, or pneumothorax. And "passive atelectasis" develops in dependent lung regions due to loss of transpulmonary pressure gradients in the anesthetized, non-ventilating patient [27].

In veterinary patients, atelectasis begins to develop during the induction of anesthesia and persists into the postoperative period, particularly in dorsal recumbency, in obese patients, and following prolonged procedures. Dependent lung regions, particularly the right middle lobe in dogs positioned in dorsal recumbency, are disproportionately affected. Sustained atelectasis creates regions of low ventilation-perfusion (V/Q) mismatch and intrapulmonary shunting, manifesting clinically as hypoxemia. Importantly, atelectatic lung tissue provides a favorable environment for bacterial colonization, potentially progressing to pneumonia, particularly in immunocompromised or critically ill patients [28,29].

Aspiration of oropharyngeal or gastric contents during or after anesthesia represents one of the most clinically significant PPCs in veterinary medicine [30]. The CEPSAF study noted that cardiovascular and respiratory causes, including aspiration events, accounted for the majority of perianesthetic deaths. Aspiration pneumonitis results from the direct chemical injury inflicted by acidic gastric contents, while aspiration pneumonia arises from the secondary bacterial colonization of lung tissue damaged by aspirated material. The right middle lung lobe is most frequently affected in dogs, owing to the anatomy of the right middle lobar bronchus relative to the trachea, which facilitates drainage of aspirated material [31].

A multicenter retrospective study identified the incidence of post-anesthetic aspiration pneumonia at 0.17% from 140,711 anesthetic events across six veterinary teaching hospitals between 1999 and 2009 [32]. Independent anesthesia-related risk factors included regurgitation during the anesthetic event and hydromorphone administration at induction. Predisposing factors for aspiration included megaesophagus, laryngeal paralysis, esophageal disease, reduced level of consciousness, and prolonged recumbency. Aspiration in anesthetized animals is particularly dangerous because the cough reflex is suppressed and the presence of an endotracheal tube, while protective of the glottis, does not prevent aspiration of material that pools above the cuff. The severity of aspiration injury correlates with both the volume and pH of the aspirated material, with acidic gastric juice producing the most severe acute lung injury [31].

Postoperative pneumonia in veterinary patients may arise de novo or as a complication of aspiration. For example, a retrospective evaluation of 707 dogs anesthetized for diagnosis or treatment of intervertebral disc disease found that postoperative pneumonia was associated with a longer duration of the first anesthetic episode and tetraparesis prior to anesthesia [28]. Inhalant anesthetic agents have been shown to impair pulmonary host defenses through inhibition of alveolar macrophage activity, suppression of mucociliary clearance, and reduction of neutrophil oxidative burst activity, collectively creating a window of immunological vulnerability during the immediate postoperative period [33,34]. Nosocomial pathogens, including *Staphylococcus*, *Escherichia coli*, *Pasteurella*, and *Klebsiella* species, are commonly implicated in postoperative veterinary pneumonia, with antimicrobial resistance an increasing concern [31].

Hypoventilation, indicating insufficient alveolar ventilation to maintain normocapnia (often defined as ETCO_2_ or PaCO_2_ greater than 45-50 mmHg in dogs and cats), is among the most frequently documented PPCs in veterinary patients recovering from general anesthesia [35]. In the recovery period, hypoventilation may result from residual opioid or inhalant effects, splinting, airway obstruction, or neuromuscular dysfunction. Sustained hypoventilation produces respiratory acidosis and, in room air, hypoxemia.

Veterinary ARDS (ARDSVet) represents the most severe form of acute lung injury and is associated with high mortality in critical patients. Updated consensus definitions for ARDSVet incorporate criteria for acute onset, risk factors, bilateral radiographic pulmonary infiltrates, exclusion of cardiogenic edema, and impaired oxygenation with severity stratified by PaO_2_:FiO_2_ ratio in both intubated and non-intubated animals [36]. Postoperative ARDS may arise from direct pulmonary causes such as aspiration, pneumonia, and pulmonary contusion, or indirect causes including sepsis, pancreatitis, and systemic inflammatory response syndrome (SIRS) [35].

Risk Factors for PORD and PPCs in Veterinary Patients

Table 1 summarizes the principal patient-, anesthetic-, and surgery-related risk factors for PORD and PPCs discussed in this section, most of which are derived from veterinary-specific data [3-5,28,37-43].

**Table 1:** Risk factors for PORD and PPCs. Shown are the common risk factors that predispose to postoperative respiratory depression (PORD) and other postoperative respiratory complications (PPCs) in veterinary surgery. PORD: postoperative respiratory depression; PPCs: postoperative respiratory complications; ASA: American Society of Anesthesiologists.

Category	Risk factor	Key finding/effect size	Ref.
Patient status	ASA grades III-V	3.26- and 4.83-fold risk of anesthetic death in dogs and cats, respectively	[3-5]
Breed/body condition	Brachycephalic conformation; obesity; advanced age	Reduced upper-airway/respiratory reserve and heightened sedative sensitivity	[37]
Pre-existing disease	Pre-anesthetic tetraparesis (cervical disease)	Significantly higher rates of postoperative pneumonia	[28]
Anesthetic factors	Prolonged anesthetic duration	Hypothermia in 63.8% of hemilaminectomy dogs; independently associated with PPC risk	[28,39,40]
Analgesic selection	Butorphanol or oxymorphone vs. hydromorphone	Statistically higher PPC rates with butorphanol/oxymorphone	[41]
Surgical factors	Emergency abdominal surgery	15-day mortality 20.7%; total complication rate 80.5%	[38,42]
Surgical approach	Laparoscopic CO_2_ pneumoperitoneum	Hypercapnia and reduced pulmonary compliance, especially with pre-existing cardiorespiratory disease	[43]

ASA physical status: ASA physical status classification is a well-validated predictor of anesthetic mortality and perioperative complications in veterinary patients. CEPSAF data demonstrated that dogs and cats classified as ASA grade III or above had 3.26- and 4.83-fold the risk, respectively, of anesthetic-related death compared with healthier counterparts [3-5].

Breed and patient characteristics: Brachycephalic breeds, including French Bulldogs, English Bulldogs, and Pugs, represent a particularly high-risk population due to anatomically compromised upper airways, reduced pharyngeal tone under anesthesia, and increased predisposition to airway obstruction during recovery [37]. These breeds are at increased risk for PORD due to the combination of reduced reserve capacity and heightened sensitivity to the sedative effects of opioids and other premedications. Obesity further compounds respiratory risk by reducing functional residual capacity, promoting dependent atelectasis, and increasing the work of breathing. Likewise, geriatric animals are disproportionately susceptible to PORD due to reduced hepatic metabolism and renal clearance of opioids, lower respiratory muscle reserve, and increased prevalence of concurrent cardiorespiratory disease.

Pre-existing conditions: Pre-existing pulmonary disease, including laryngeal paralysis, tracheal collapse, pulmonary fibrosis, and pneumonia, substantially elevates the risk of PPCs, as does cervical spinal cord disease in dogs. For example, a study found that dogs with pre-anesthetic tetraparesis had significantly higher rates of postoperative pneumonia, which was attributed to diaphragmatic and accessory muscle dysfunction and impaired cough reflexes [28]. Pre-operative hypoalbuminemia, commonly encountered in critically ill veterinary patients, was associated with increased postoperative mortality and pulmonary edema risk in a study of emergency laparotomy [38].

Anesthetic and analgesic factors: Anesthetic duration is a consistent risk factor for PPCs across veterinary studies, with longer anesthetic duration identified as an independent factor associated with increased PPC risk [28,39]. Prolonged anesthesia results in cumulative inhalant and opioid exposure, progressive atelectasis from sustained recumbency, and impaired thermoregulation, all of which converge to compromise postoperative respiratory function. In a study of dogs undergoing hemilaminectomy, hypothermia was found to be the most common intraoperative complication, developing in 143 of 224 dogs (63.8%), with anesthetic duration significantly associated with both hypothermia and the need for mechanical ventilation [40].

Opioid selection also influences the risk for PPC development. For example, the CEPSAF-derived laparotomy data demonstrated that use of butorphanol or oxymorphone for postoperative analgesia was associated with statistically higher PPC rates compared to hydromorphone, suggesting that MOR agonist efficacy may be protective against hypoventilation secondary to pain-induced splinting [41]. However, the paradox of opioid analgesia - reducing pain and splinting while simultaneously depressing respiratory drive - requires careful individual titration. Regurgitation during anesthesia, which carries the risk of aspiration, occurred in approximately 11 of 224 (4.9%) dogs undergoing hemilaminectomy and was an independent predictor of post-anesthetic aspiration pneumonia in a large multicenter study [32,40].

Surgery factors: The site and type of surgery are contributing determinants of PPC risk [42]. Upper abdominal surgery and thoracotomy impose the greatest respiratory risk due to diaphragmatic dysfunction, pain-induced splinting, and direct pulmonary trauma. In dogs, exploratory laparotomy for biliary or septic peritonitis was identified as an independent risk factor for PPCs, likely due to the combination of systemic inflammation, direct diaphragmatic irritation, and increased aspiration risk [39].

Not surprisingly, emergency surgery significantly elevates PPC risk compared with elective procedures, as documented in dogs undergoing laparotomy, and in a retrospective analysis of 82 dogs undergoing emergency abdominal surgery, in which an overall 15-day mortality rate of 20.7% and a total complication rate of 80.5% were reported, with pulmonary complications including aspiration pneumonia and pulmonary thromboembolism contributing to deaths [38]. Neurosurgical patients, particularly those with cervical myelopathy requiring prolonged anesthetic events and postoperative recumbency, constitute another high-risk group.

Laparoscopic procedures, while associated with lower PPC rates than equivalent open surgical procedures in human medicine due to reduced pain and less diaphragmatic impairment, carry specific respiratory risks in veterinary patients related to CO_2_ pneumoperitoneum. Insufflation with CO_2_ induced hypercapnia, reduced pulmonary compliance, and, in patients with pre-existing cardiorespiratory disease, clinically significant cardiorespiratory depression [43].

Diagnosis and Monitoring

The AAHA 2020 Anesthesia and Monitoring Guidelines emphasize that between 47% and 60% of all anesthetic-related deaths in dogs and cats, respectively, occur during the postoperative period, with the majority occurring within the first three hours [19]. This suggests that monitoring practices during the recovery phase should be as rigorous as those maintained during the phase of anesthesia. Pulse oximetry is recommended in all heavily sedated or anesthetized small animal patients, and evidence from the CEPSAF study associates postoperative use with decreased risk of peri-anesthetic mortality.

ETCO_2_ monitoring via capnography provides some real-time assessment of alveolar ventilation and provides practical indication of hypoventilation and apnea in intubated patients. In the human literature, continuous capnography has been associated with reductions in opioid-induced respiratory rescue events [44]. Capnography should be maintained during the early recovery period in high-risk patients, including brachycephalic breeds, obese animals, patients with pre-existing pulmonary disease, and those receiving continuous opioid rate infusions. In the PRODIGY trial - a landmark prospective study of 1,335 postoperative human patients on opioids - continuous capnography and pulse oximetry detected OIRD events in 46% of monitored patients, a rate that would be undetectable by intermittent nurse observations alone [45]. While a comparable veterinary-specific study has not been conducted, the physiological principles directly apply.

Arterial blood gas analysis provides definitive assessment of arterial oxygenation (PaO_2_), ventilation (PaCO_2_), and acid-base status and should be performed in any patient demonstrating clinical signs of respiratory compromise, in those at high risk for PPCs, or, arguably, in all patients. Normal PaO_2_ in dogs and cats breathing room air is approximately 85-100 mmHg; values below 80 mmHg are commonly used as an indication of hypoxemia. Normal PaCO_2_ is 35-45 mmHg; values above 50 mmHg are considered clinically significant hypoventilation. Thoracic radiography remains the primary imaging modality for confirming PPCs, including atelectasis, pneumonia, and pulmonary edema, though computed tomography provides superior sensitivity and spatial resolution, particularly for bronchiectasis, pulmonary foreign bodies, and early interstitial disease [31].

PORD may manifest as reduced respiratory rate (below eight breaths per minute in dogs or 10 breaths per minute in cats during recovery), reduced tidal volume, paradoxical thoracoabdominal movement, excessive sedation, failure to maintain sternal recumbency, prolonged extubation time, and progressive oxygen desaturation on pulse oximetry. Severe OIRD may present with frank apnea, deep coma, miosis, and cardiovascular compromise [6]. PPCs may present with tachypnea, increased respiratory effort, cough, pyrexia, mucopurulent nasal discharge, cyanosis, reduced lung sounds on auscultation, or crackling sounds indicative of alveolar fluid. Animals with ARDS present with progressive hypoxemia refractory to supplemental oxygen, bilateral pulmonary infiltrates on imaging, and signs of systemic inflammatory disease [36].

Prevention Strategies

Systematic preoperative risk stratification using the ASA physical status score, combined with a thorough history, physical examination, and targeted diagnostics, forms the cornerstone of PPC prevention [46]. Patients with known risk factors for aspiration, including megaesophagus, esophageal dysmotility, laryngeal paralysis, or decreased mentation, should be fasted appropriately and considered for prokinetic prophylaxis. The potent neurokinin NK-1 receptor antagonist maropitant has antiemetic efficacy in dogs and may reduce perioperative vomiting and the associated risk of aspiration [47]. Pre-oxygenation for a minimum of three minutes prior to anesthetic induction increases hemoglobin oxygen saturation reserves, providing up to six minutes of additional safe apnea time, and is particularly critical in patients with airway disease or breathing difficulties, as highlighted in the AAHA 2020 guidelines.

Preoperative physiotherapy and respiratory exercises, including nebulization and coupage, are indicated in patients with pre-existing pulmonary secretion accumulation or atelectasis. Patients with laryngeal paralysis should be stabilized medically before surgery where possible, and thoracic radiographs should be performed to evaluate for pre-existing aspiration pneumonia, which affects outcome in this population [48].

Protective lung ventilation (PLV) strategies, developed originally in the context of human ARDS management, are increasingly advocated for intraoperative ventilation in veterinary patients. PLV employs tidal volumes of 6-8 mL/kg, positive end-expiratory pressure (PEEP) of 5-8 cm H_2_O to maintain alveolar recruitment, and plateau airway pressures below 30 cm H_2_O to minimize ventilator-induced lung injury. PEEP reduces the formation of dependent atelectasis, improves V/Q matching, and may reduce postoperative hypoxemia [49]. Recruitment maneuvers, consisting of a sustained inflation to 30-40 cm H_2_O for 20-30 seconds, can transiently reverse established atelectasis but should be performed with hemodynamic monitoring in place given their potential to reduce venous return and cardiac output.

Minimizing total opioid dose through multimodal analgesia is helpful for PORD prevention. Locoregional anesthetic techniques, including epidural, intercostal, paravertebral, and brachial plexus blocks, are effective in reducing systemic opioid use and the resultant respiratory depression risk. NSAIDs, ketamine constant rate infusions (CRIs), lidocaine CRIs, and dexmedetomidine CRIs provide adjunctive analgesia and inhalant-sparing effects without the respiratory depression associated with opioids [25]. Positioning strategies during recovery, including prompt transition to sternal recumbency, facilitate drainage of dependent airway secretions, improve functional residual capacity, and restore more normal physiological respiratory mechanics.

Maintenance of normothermia is helpful, as hypothermia prolongs drug metabolism, impairs coagulation, and increases oxygen consumption during shivering in recovery, all of which can exacerbate respiratory compromise [50]. Active warming should be implemented throughout anesthesia using forced-air warming devices, warm water blankets, insulating materials, and warmed intravenous fluids.

The postoperative period requires continued vigilance proportional to the risk profile of the patient. High-risk patients should remain under continuous monitoring in a dedicated recovery area staffed by trained personnel. Supplemental oxygen via face mask, flow-by oxygen, or nasal cannula should be administered to any patient demonstrating signs of hypoventilation or hypoxemia [51]. Patients who fail to achieve adequate ventilation spontaneously should receive assisted or controlled ventilation via reintubation. Sternal recumbency should be provided as promptly as possible following recovery from anesthesia, as lateral recumbency increases compressive atelectasis of the dependent lung.

Naloxone, the primary opioid antagonist, should be immediately available in all clinical settings where opioids are administered. In the setting of life-threatening OIRD, naloxone should be administered intravenously in dogs and cats, titrated to effect to reverse respiratory depression without precipitating abrupt withdrawal, pain reinstatement, and cardiovascular stress [52]. Partial reversal is preferable to complete antagonism because the latter removes the analgesic component and may cause acute sympathomimetic crisis. The plasma half-life of naloxone is shorter than that of most opioids, necessitating repeat dosing or a CRI in cases involving longer-acting opioids such as buprenorphine or morphine.

For patients developing infectious PPCs, early antimicrobial therapy guided by cytological and culture results from bronchoalveolar lavage or tracheal wash is indicated. Physiotherapy, including nebulization with saline or mucolytics, coupage, and assisted ambulation, promotes secretion clearance and reduces the progression of atelectasis to pneumonia [53]. In patients with ARDS, lung-protective ventilation, treatment of the underlying cause, and judicious fluid therapy to avoid exacerbating pulmonary edema are the pillars of the standard of care.

Treatment of established PORD and PPCS

When severe OIRD is diagnosed or strongly suspected in a postoperative patient, immediate action is required. Supplemental oxygen should be provided, and if respiratory effort is inadequate, manual positive pressure ventilation via face mask or endotracheal tube should be initiated. Naloxone remains the definitive pharmacological treatment for OIRD. As noted, careful titration is essential: in dogs and cats, incremental intravenous doses of 0.002-0.01 mg/kg are administered every 2-3 minutes until the respiratory rate and depth are adequate while preserving as much analgesia as possible. In cases where reversal precipitates severe pain, transition to non-opioid analgesia, including ketamine, NSAIDs, or regional nerve blocks, should be rapidly implemented [11].

Alternative respiratory stimulants, termed "agnostic" respiratory stimulants, represent an area of active investigation in human anesthesia. Doxapram, a non-specific respiratory stimulant acting on peripheral chemoreceptors and brainstem centers, has been used in veterinary medicine as an adjunct in neonatal resuscitation and in patients with respiratory depression of varying etiology. Its use in PORD is primarily supportive, bridging the period until opioid concentrations decline or until naloxone achieves effect. However, doxapram has a brief duration of action (5-10 minutes), and it may cause cardiovascular stimulation.

The management of aspiration pneumonia in veterinary patients involves supplemental oxygen therapy, antimicrobial treatment, airway physiotherapy, and supportive care [54]. Thoracic radiography should be performed as early as possible when aspiration is suspected, recognizing that radiographic changes may lag up to 24 hours behind clinical signs. Initial antimicrobial therapy should provide broad-spectrum coverage against gram-negative enteric organisms and anaerobes, pending culture and sensitivity results from airway sampling [55]. Aminoglycosides, fluoroquinolones, and beta-lactam antibiotics in combination are commonly employed. The benefit of corticosteroid administration in aspiration pneumonitis remains questionable and is not currently recommended as routine practice [56].

The placement of an esophagostomy or nasogastric tube in patients with prolonged aspiration pneumonia facilitates enteral nutrition, which supports immune function and mucosal integrity, and avoids the morbidity of parenteral nutrition [57]. Patients with severe lung injury from aspiration may require mechanical ventilation with lung-protective settings and management in a specialist intensive care environment.

Regardless of the specific PPC etiology, a standardized supportive care approach applies [58]. Regular position changes, at minimum every 2-4 hours, reduce the progression of dependent atelectasis in recumbent patients. Nebulization with isotonic saline loosens secretions and improves mucociliary clearance, while gentle coupage facilitates movement of loosened secretions toward the large airways. Early ambulation, where patient status permits, promotes diaphragmatic function, reduces atelectasis, and improves respiratory reserve. Nutritional support should not be delayed in critically ill patients, as malnutrition independently impairs respiratory muscle function and immune competence [59].

Special considerations in selected patient populations

Brachycephalic obstructive airway syndrome (BOAS) constitutes a distinct and high-risk entity in perioperative respiratory management [60]. Affected dogs, including French Bulldogs, English Bulldogs, and Pugs, and brachycephalic cats such as Persians, have special anatomy, including stenotic nares, an elongated soft palate, a hypoplastic trachea, and everted laryngeal saccules that collectively impair upper airway patency. Under anesthesia, loss of upper airway muscle tone produces airway obstruction proportionally more severe than in other patients. During recovery, as anesthetic depth lightens, upper airway obstruction frequently manifests as inspiratory stridor, respiratory distress, paradoxical chest wall motion, and desaturation. It is recommended that prolonged extubation should be avoided; instead, these patients should be maintained intubated until they are sufficiently awake to actively resist the tube. Post-extubation oxygen supplementation and patient positioning in sternal recumbency are advised. Surgical correction of correctable BOAS components prior to any elective anesthesia reduces but does not eliminate perioperative risk. Implementation of special protocols decreases the incidence of postoperative regurgitation in brachycephalic dogs [61].

Cats warrant specific consideration in the context of PORD and PPCs due to species-specific physiological differences and pharmacological responses. The CEPSAF study established that cats have a higher baseline anesthetic mortality than dogs (0.24% for healthy cats vs. 0.11% for healthy dogs), with 61% of cat deaths occurring postoperatively compared with 47% in dogs [4]. Feline opioid pharmacology differs from that of dogs: cats exhibit a reduced conjugation metabolism capacity for morphine, increased sensitivity to opioid-induced dysphoria, and potentially different MOR density distributions. Buprenorphine is considered the opioid of choice for postoperative analgesia in cats due to its high analgesic efficacy, relatively low respiratory depression risk as a partial agonist, and its effective mucosal absorption, facilitating non-invasive administration. Feline ARDS definitions and management follow similar principles to canine patients, though specific veterinary data are more limited. Cats presenting with severe pulmonary contusions, pyothorax, or severe aspiration events may develop acute lung injury requiring ventilatory support. The smaller body size of most cats makes mechanical ventilation technically feasible but necessitates careful tidal volume calculations using lean body weight in order to avoid volutrauma.

While this review focuses primarily on small animal patients, it is appropriate to mention that large animal anesthesia carries its own substantial respiratory risks in the postoperative period. Horses anesthetized in dorsal recumbency experience marked reduction in functional residual capacity, significant dependent atelectasis, and post-anesthetic myopathy, which can impair the ability to maintain sternal recumbency during recovery. Post-anesthetic pulmonary edema, fractured ribs during recovery, and aspiration events during induction and recovery constitute important large animal PPCs. The equine perioperative anesthetic mortality rate has been estimated at approximately 1% in referral populations, substantially higher than in companion animals, with respiratory causes contributing significantly [62].

Discussion

Several important gaps remain in the veterinary-specific evidence base for PORD and PPCs. The development of validated risk prediction tools analogous to the PRODIGY score for human patients could facilitate prospective identification of high-risk veterinary patients and guide targeted monitoring strategies. Point-of-care lung ultrasound has emerged as a sensitive, non-invasive imaging modality for detecting atelectasis and pulmonary consolidation in awake and anesthetized veterinary patients and warrants further investigation as a bedside monitoring tool in the recovery suite.

Pharmacological innovations, including peripherally acting MOR antagonists and opioid-sparing analgesic strategies such as liposomal bupivacaine, nerve growth factor antibodies, and sustained-release NSAIDs, may further reduce PORD risk without compromising analgesia. The application of lung-protective ventilation protocols in routine veterinary anesthetic practice, rather than only in critical cases, suggests the value of prospective veterinary studies to define optimal intraoperative tidal volume, PEEP, and recruitment maneuver protocols by species and body weight range. A new pharmacologic approach being investigated for countering PORD is inhibition of BK channels in the carotid bodies. This peripheral mechanism yields rapid response (no need to cross the blood-brain barrier) without CNS effects and is independent of the drug-depressant cause [63]. Existing pharmacologic options for reversing PORD remain imperfect: naloxone is effective only against opioid-mediated respiratory depression and simultaneously reverses analgesia (with precipitated withdrawal), while doxapram is non-specific, short-acting, and can cause cardiovascular effects. This gap underscores a clinical need for an "agnostic" respiratory stimulant, i.e., an agent capable of restoring adequate ventilation regardless of the underlying cause (opioid, propofol, anesthetic, etc.) and without reversing analgesia or requiring identification of the specific causative drug; peripherally acting BK-channel inhibitors of the type described above represent one such investigational strategy, but veterinary-specific safety and efficacy data are currently lacking and are needed before clinical adoption can be considered.

Systematic postoperative monitoring protocols, including standardized respiratory assessment scores analogous to the Ramsay Sedation Scale used in human medicine, could improve consistency of recovery monitoring across veterinary institutions. Adoption of continuous pulse oximetry combined with capnography for high-risk recovery patients, supported by automated alerting systems, represents a feasible near-term improvement in perioperative safety that could significantly reduce the incidence of undetected PORD events.

## Conclusions

PORD and PPCs represent important and incompletely mitigated causes of postoperative morbidity and mortality in veterinary patients. The CEPSAF data establish that the postoperative period - particularly the first three hours - is the most critical phase and that respiratory and cardiovascular causes dominate perioperative deaths in dogs and cats. Comprehensive risk stratification, anesthetic protocol optimization, including multimodal analgesia and lung-protective ventilation, meticulous perioperative monitoring, and prompt recognition and treatment of PORD and PPCs are essential to improving outcomes. As the veterinary evidence base continues to grow, parallel adoption of best practices from human anesthesiology - validated against species-specific data - should help further reduce the gap between human and veterinary anesthetic mortality rates.
